# Findings on e-cigarette flavors and their implications for human health: a systematic review

**DOI:** 10.3389/fpubh.2026.1807715

**Published:** 2026-04-30

**Authors:** Nayeli Itzel Vázquez-López, Ramcés Falfán-Valencia, Gloria Pérez-Rubio

**Affiliations:** Pneumogenomics Laboratory, Instituto Nacional de Enfermedades Respiratorias Ismael Cosío Villegas, Mexico City, Mexico

**Keywords:** animal models, e-cigarettes, e-flavors, human studies, tobacco smoking, vaping

## Abstract

**Objectives:**

This systematic review evaluated the scientific evidence on the effects of flavors in electronic cigarettes in animal models and their impact on human health, aiming to understand the potential risks associated with their use.

**Methods:**

A PubMed search was conducted using MeSH terms such as “e-cigarettes AND flavor AND nicotine AND e-liquid AND cytotoxicity AND cellular damage and health AND adolescent AND young people.” We selected original studies that evaluated tissue damage, inflammation, oxidative stress, and DNA damage in animal models, as well as health outcomes in humans. *In vitro* studies, studies with conflicts of interest related to the tobacco industry, and studies that did not meet our objectives were excluded.

**Results:**

Fruity, menthol/mint, and sweet flavors increase nicotine consumption and preference for e-cigarettes in animal models and are also linked to inflammation, cellular damage, and cardiovascular changes. In humans, these flavors have been associated with respiratory symptoms, reduced lung function, and DNA damage in oral cells. Neurological effects, such as activation of reward circuits, have also been observed.

**Conclusion:**

The evidence available to date suggests that these products can be as harmful to health as combustible cigarettes.

**Systematic Review Registration:**

https://www.crd.york.ac.uk/PROSPERO/view/CRD420261346170.

## Introduction

Electronic cigarettes (e-cigs) are battery-operated devices that transform a solution called e-liquid into an aerosol. This is produced by an open- or closed-system electric heating element that activates when the user inhales air through the device (1). Closed system devices are characterized by pre-filled liquid-in cartridges or capsules that cannot be refilled and, in most cases, feature a preset voltage of 3.7 volts (2). In contrast, open systems allow users to fill the device with the liquid of their choice; furthermore, they do not have a preset voltage. Higher voltage settings can generate resistance temperatures ranging from 100°C to over 1,000°C (3). Another point to consider is that toxic metals such as chromium, copper, and lead are released as the device ages over time (4). In addition to heavy metals, there are chemical compounds that can cause adverse health effects; these have been termed “potentially harmful and hazardous components” (HPHCs). These include carbonyl compounds (formaldehyde, acetaldehyde, and acrolein), volatile organic compounds (VOCs, benzene, toluene, and styrene), and heavy metals (lead, arsenic, nickel, and iron) (5). The latest fourth-generation e-cigarettes incorporate free organic acids, such as benzoic acid and levulinic acid, to achieve a lower pH, reduce the harshness of vaping, and enhance the kinetics of aerosolized nicotine (6). E-liquids currently available on the market contain base vehicles such as propylene glycol (PG) and vegetable glycerin (VG). According to studies conducted on C57BL/6 mice, the thermal degradation of e-liquids containing PG and VG generates volatile organic compounds (VOCs) that may cause vascular damage. Compounds identified included formaldehyde, acetaldehyde, acetone, and propionaldehyde. PG produces more formaldehyde, whereas VG produces more acrolein. These aldehydes can lead to lung irritation and endothelial dysfunction (7).

Aerosol of PG/VG (50%/50%) *in vitro* models increased the expression of inflammatory markers (IL-6, IL-8, and MMP-9) and mucins (MUC5AC and MUC5B) (8). Exposure to PG aerosols reduced cell viability by 50%, while glycerin aerosols reduced it by 63% (9).

C57BL/6J mice exposure to PG/VG increased the levels of inflammatory cytokines (keratinocyte chemoattractant, IL-6, and monocyte chemoattractant protein-1) in bronchoalveolar lavage fluid (BALF), suggesting lung damage (10).

The addition of flavors to e-cigarettes has resulted in adolescents and young adults being the primary consumers of these electronic devices (11). Reports indicate that users have started vaping as early as age 12 (12). Fruity flavors are among the most consumed, accounting for 43.6%, followed by sweets and desserts at 27.6% (13).

Caucasian adolescents and young adults using fruity, mint, or menthol-flavored e-liquids with high nicotine concentrations (≥5%) have shown higher dependence, increased vaping frequency, and lower intentions to quit, suggesting a dose-response relationship between nicotine strength and addiction (14). Packaging featuring flavor descriptions was seen as more appealing and elicited greater receptivity than unflavored devices (15).

Those who favor these flavors also tend to use them more frequently (18.71 days vs. 12.18 days) and start vaping at a younger age (13.6 years vs. 14.2 years) (16). The prevalence of consumption by sex indicates that men prefer devices with a larger capacity (>2 mL) and higher nicotine concentration (>20 mg/mL); women favor more discreet options, such as pen-type devices (17).

According to the 2022 National Continuous Health and Nutrition Survey in Mexico, the prevalence of e-cigarette use among Mexican adolescents was 2.6%; among these, 2.1% were women and 3.0% were men (18).

*In vitro* models, substances present in e-cigarette liquids, such as flavorings, have been shown to increase oxidative stress, inflammation, and cytotoxicity—phenomena linked to the development of lung diseases such as COPD and lung cancer. This evidence suggests mechanisms of interest that could be involved in diseases associated with e-cigarette use.

However, these findings must first be tested in animal models and subsequently evaluated to determine if the same occurs in humans.

**Figure 1:**
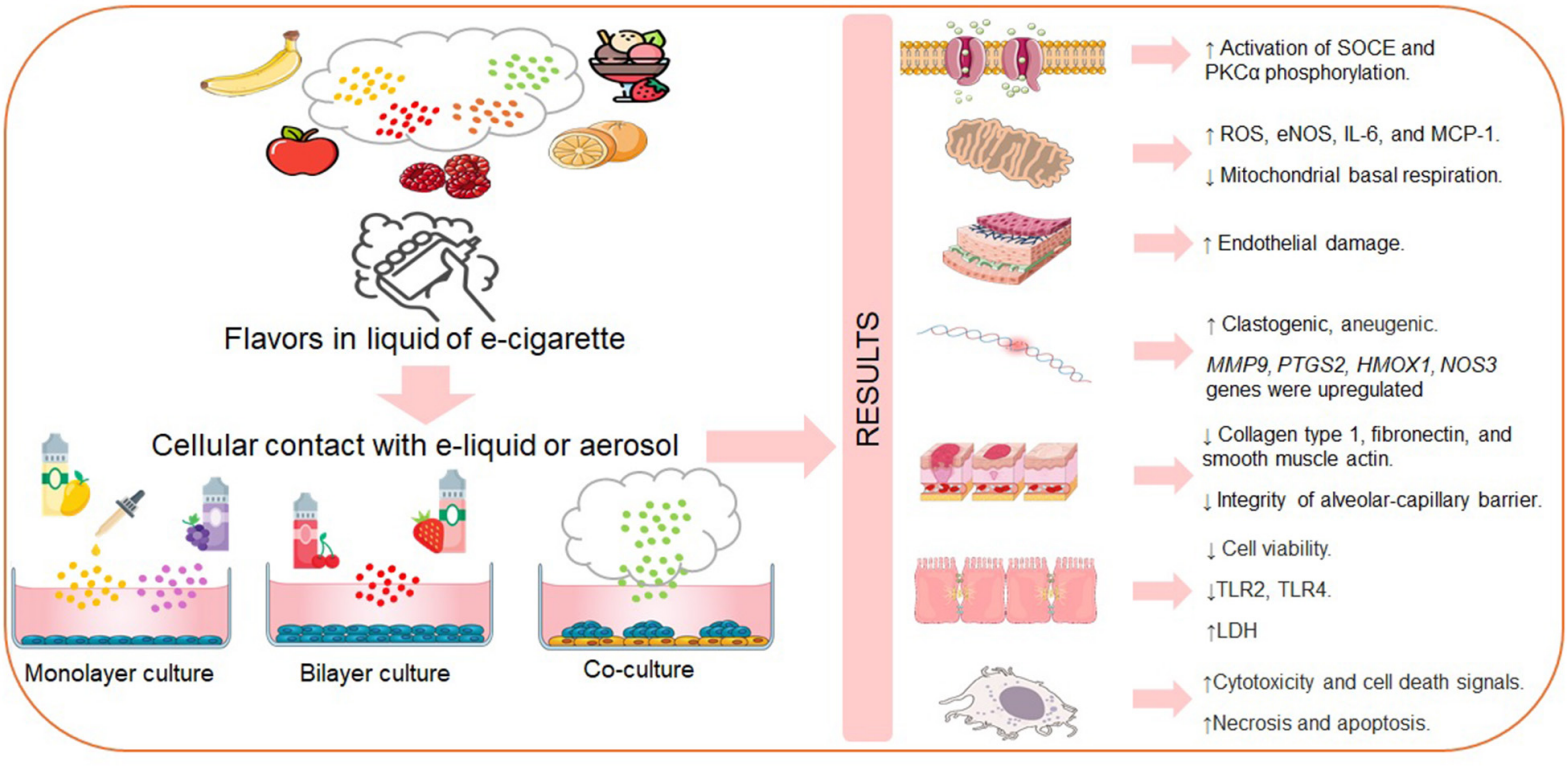
Assessing the damage caused by the flavors of e-cigarette liquids *in vitro* models. SOCE, Store-operated Ca2+ entry. PKCα protein kinase C; ROS, reactive oxygen species; NOS, nitric oxide species; IL-6, Interleukin-6; MCP-1, Monocyte chemoattractant protein-1; MMP9, Matrix metalloproteinase-9; PTGS2, Prostaglandin-endoperoxide synthase 2; HMOX1, Heme oxygenase 1; NOS3, Nitrosamine O-S methyltransferase; TLR2, Toll-like receptor 2; TLR4, Toll-like receptor 4; LDH, Lactate dehydrogenase. Made with: https://www.flaticon.com, https://bioicons.com, https://bioart.niaid.nih.gov/, and https://smart.servier.com/.

Mechanisms such as oxidative stress, cytotoxicity, inflammation, mitochondrial dysfunction, and DNA damage have been documented in cell models and appear to be influenced by factors including liquid dosage, the chemical composition of flavorings, and the type of device used. Cellular models provide a foundation for future research in animal models and long-term clinical studies aimed at evaluating the health consequences of e-cigarette use (Figure 1). Given the growing popularity of e-cigarette flavors and the lack of clear scientific evidence on their health effects, this systematic review aims to assess the available scientific evidence on the impact of e-cigarette flavors in animal models and findings from human studies. The goal is to provide a comprehensive understanding of the potential risks associated with the use of flavored e-cigarettes.

## Materials and methods

A search of scientific literature was conducted using the “PubMed” database (19) and the Cochrane Library (20).

The following Medical Subject Headings (MeSH) terms were used: “e-cigarettes AND flavor AND nicotine AND e-liquid AND cytotoxicity AND cellular damage and health AND adolescent AND young people.” We included publications from 2020 to 2025 because fourth-generation electronic cigarettes appeared on the market in 2020 (21). Studies in all languages were included. The selection criteria included original articles evaluating e-cigarette flavors, tissue damage caused by the use of electronic nicotine delivery systems (ENDS), inflammation and cellular damage, and DNA damage in animal models, as well as health outcomes in humans. Exclusion criteria were conflicts of interest with the tobacco industry, *in vitro* studies, *in silico* models, and studies using cannabis cigarettes or tobacco flavors, hookahs, nicotine pouches, and waterpipes. We also excluded studies on health policies or perceptions of vaping.

Literature selection was performed following the PRISMA (Preferred Reporting Items for Systematic Reviews and Meta-Analyses) 2020 guidelines (22). Two reviewers independently performed the study selection (NIVL and GPR). Discrepancies were resolved through consensus with a third reviewer (RFV). A risk-of-bias assessment was performed. SYRCLE (for animal studies) (23) and the Newcastle-Ottawa Scale (for observational studies in humans) (24) were used.

## Results

The search returned 430 articles that met the selection criteria. Four hundred and sixteen articles were excluded because they were duplicate records or not original research articles (letters to the editor, commentaries, reviews), presented potential conflicts of interest, or failed to disclose results or methodology. Studies on cannabis cigarettes or waterpipes were also excluded, along with those that did not meet our objectives (studies evaluating consumption patterns, epidemiology, flavor preferences in youth, among others). Thirteen papers were included for analysis (Supplementary Figure 1). Of these, nine were conducted in animal models, and, according to the SYRCLE tool, the risk of bias in these studies ranged from low to uncertain. It is worth noting that there was heterogeneity among the biological models analyzed; however, to date, this is the only available evidence (Supplementary Table 1). For studies with direct evidence in humans, only 4 could be included; of these, 25% were of moderate quality, while the rest were of high quality according to the Newcastle–Ottawa scale (Supplementary Table 2). The protocol was registered in PROSPERO (CRD420261346170. https://www.crd.york.ac.uk/PROSPERO/view/CRD420261346170).

### Animal models

The use of flavors increases the appeal and preference for e-cigarette use, especially among young people. Using animal models, researchers have demonstrated that flavors enhance consumer acceptance compared to unflavored vehicles. The addition of flavor additives to e-cigarettes improves chemosensory properties and influences nicotine intake (Table 1).

**Table 1:** Studies on the flavors in electronic cigarettes using animal models.

PG/VG (%/%)	Exposition	Flavor	Dose of flavor	Nicotine concentration	Time of exposure	Findings	Reference
50/50	Liquid	Fruit	16.1-17.7 mg/dL	30, 50, 75, 100, and 200 μg/mL	1 week	Mice consumed more e-liquid flavored with fruit and containing nicotine than nicotine alone.	Wong et al. (25)
50/50	Aerosol	Strawberry	2.5% and 10%	2.5, 10, and 50 mg/mL	25 min (5 days twice a day)	The mice showed a clear preference for strawberry-flavored e-cigarette vapes compared to nicotine-only versions.	Patten et al. (27)
30/70	Aerosol	Menthol	NS	5%	2 weeks	There was increased platelet aggregation and enhanced thrombosis. Platelets from exposed mice showed increased ATP and P-selectin expression.	Ramirez et al. (30)
70/30	Aerosol	French Vanilla	0.035 mg/puff	Without	2 h a day, 7 days a week, for 6 weeks	A slight increase in NK cell and dendritic cell populations (CD11b+, CD11c+) was observed. There was an increase in Hpx expression, a gene that helps protect against oxidative damage and influences the anti-inflammatory functions of high-density lipoproteins.	Szafran et al. (31)
30/70	Aerosol	Vanilla custard, apple jax, Hawaiian POG	0.075, 0.15, 0.375, and 0.75 puff/mL	6 mg/mL	2 h per session, 5 days per week, for 10 weeks	Disrupted autonomic balance in mice, with both time-domain and frequency-domain measures showing reduced parasympathetic activity. Mice exposed to vanilla custard-flavored vaping exhibited increased susceptibility to sustained ventricular tachycardia.	Abouassali et al. (32)
20/80	Aerosol	Raspberry	3%	6, 12, 18, 24, and 36 mg/mL	1 h	Exposure to raspberry flavor can help mitigate the potential aversive effects of aerosolized nicotine.	Maeusich et al. (26)
50/50	Aerosol condensate (injected)	Cinnamon	2% and 5%	The e-liquid was purchased from the online shop	Injected in embryonic days 9 and 17	Apoptotic nuclei were prominent in scleral cartilage cell nests. Additionally, the ganglion cell layer exhibited focal areas with numerous vacuolations.	Alshareef et al. (28)
50/50	Liquid	Mint, menthol	0.075% and 0.025%	3% (commercial pod-moods)	2 days	Exposure to e-cigarette liquid is extremely harmful to the development of zebrafish. The survival thresholds were established at 0.075% for Menthol and 0.025% for Mint-flavored e-liquids. Exposure to Mint e-liquid triggered a pro-inflammatory response in zebrafish embryos, causing neutrophils to migrate from the caudal hematopoietic tissue.	Onyenwoke et al. (29)
NS	Liquid	Berries, cereal and cream, vanilla ice cream, birthday cannoli	1:100 dilution	3–6 mg/mL	48–72 h post-fertilization	Craniofacial abnormalities are shown, including midface narrowing, lens protrusion, and clefts in the primary palate and the roof of the oral cavity.	Dickinson et al. (33)

PG, propylene glycol; VG, vegetal glycerin; NS, not specified.

Male adult mice (8-week-old, C57BL/6J) were subjected to a two-bottle choice test, presented with four options: nicotine-containing fruit-flavored e-liquid vs. water, nicotine-containing tobacco-flavored e-liquid vs. water, nicotine-free fruit-flavored e-liquid vs. water, and nicotine-free tobacco-flavored e-liquid vs. water. The mice showed greater consumption and preference for nicotine-containing fruit-flavored e-liquid compared to nicotine alone, suggesting that certain flavors may enhance nicotine consumption (25). The previous finding is reinforced by a study in which 70-day-old male Sprague-Dawley rats were first trained in sucrose self-administration and then in aerosol self-administration. The aerosol contained nicotine (0, 6, 12, 18, 24, or 36 mg/mL) along with 3% raspberry flavor. The results indicated that exposure to raspberry flavor may help mitigate the potential aversive effects associated with aerosolized nicotine (26).

In adolescent C57BL/6J mice (human equivalents aged 12–18 years), the effect of a strawberry additive in e-liquids on nicotine reward and exposure was examined. Nicotine was delivered via aerosol at concentrations of 2.5, 10, and 50 mg/mL. The strawberry additive was added at concentrations of 2.5% and 10%. To assess reward, a conditioned place preference paradigm was used. Systemic exposure to nicotine was assessed by measuring plasma cotinine levels 30 min after exposure. Mice exposed to strawberry-containing nicotine vapors exhibited higher plasma cotinine levels, indicating that the strawberry additive increased the attractiveness of the nicotine vapors (27).

Cinnamon is among the most widely consumed flavors. *In vitro* studies have shown that it decreases the number of viable cells, damages DNA, and increases ROS production. In chicken embryos, a significant decrease in the apoptosis-related gene *CASP3* was observed, with a 213-fold decrease at a 2% cinnamon concentration and a 21,480-fold decrease at a 5% concentration on embryonic day 9. Histological analysis revealed disorganization of retinal layers, vacuolization, and apoptotic cell death. In retinal layers such as the inner nuclear and ganglion cell layers, multiple voids and cells with pyknotic nuclei (indicative of apoptosis) were observed. These findings suggest that flavorings, such as cinnamon, induce oxidative stress and apoptosis in the retina (28).

Another popular flavor is mint, and its derivatives. The toxicity of the menthol- and mint-flavored pod-mods was evaluated in zebrafish embryos. Doses higher than 0.075% and 0.025% (menthol and mint, respectively) were lethal to embryo survival, induced proinflammatory response, and triggered neutrophil migration. In the mouse model, the condensed vapor was administered intranasally (10 μL/day) for 5 weeks to simulate chronic exposure. Chronic exposure, in combination with MHV-A59 coronavirus infection, caused a significant increase in BALF of the proinflammatory cytokine IL-6 (from 20 pg/mL in controls to 60 pg/mL in infected mice exposed to mint). Thickening of the alveolar walls and infiltration of inflammatory cells in the lungs were also observed (29).

In a 2-week exposure model in mice (70 puffs daily), the pod-mod with menthol flavor significantly increases the risk of thrombosis and platelet activation. Increased cotinine levels, reduced bleeding time (35 s (s) vs. 295 s, *p* < 0.001), and arterial occlusion (14 s vs. 200 s, *p* < 0.01) were observed, indicating a greater propensity for thrombotic events (30).

Another flavor popular among young people is French vanilla. It contains three main flavoring compounds: ethyl vanillin, vanillin, and maltol. Chronic exposure of C57BL/6 female mice to French vanilla aerosol for 6 weeks results in increased pulmonary NK cell numbers, elevated pulmonary gene expression of Ak4 and Hpx, and increased levels of IgG1 in BALF. The increased lung tissue resistance in these mice is a multifactorial event that may involve changes in tissue compliance, viscosity, and cellular infiltration (31).

Another similar flavor is vanilla cream, which contains aldehyde compounds. In mice exposed to vaping for 10 weeks, cardiac parasympathetic activity decreased, leading to sympathetic dominance and, consequently, sympathovagal imbalance. Cardiac electrophysiological instability was also observed, potentially leading to arrhythmogenesis (32).

E-cigarettes contain teratogenic compounds. The effects of e-liquids and the flavoring compound vanillin on craniofacial development were studied in *Xenopus laevis* embryos. Early embryos were exposed to vaporized extracts (aerosols) and pure e-liquids with dessert flavors, including Berries, Cereal and Cream, and Vanilla Ice Cream. The exposure resulted in orofacial clefts, reduced ethmoid cartilage, and shortening of the mandibular muscles. Genes involved in the retinoic acid pathway, such as *aldh1a2, crabp1, crabp2, rarg*, and *cyp26c1*, exhibited altered expression, suggesting a disruption in retinoic acid synthesis and signaling (33).

**Figure 2:**
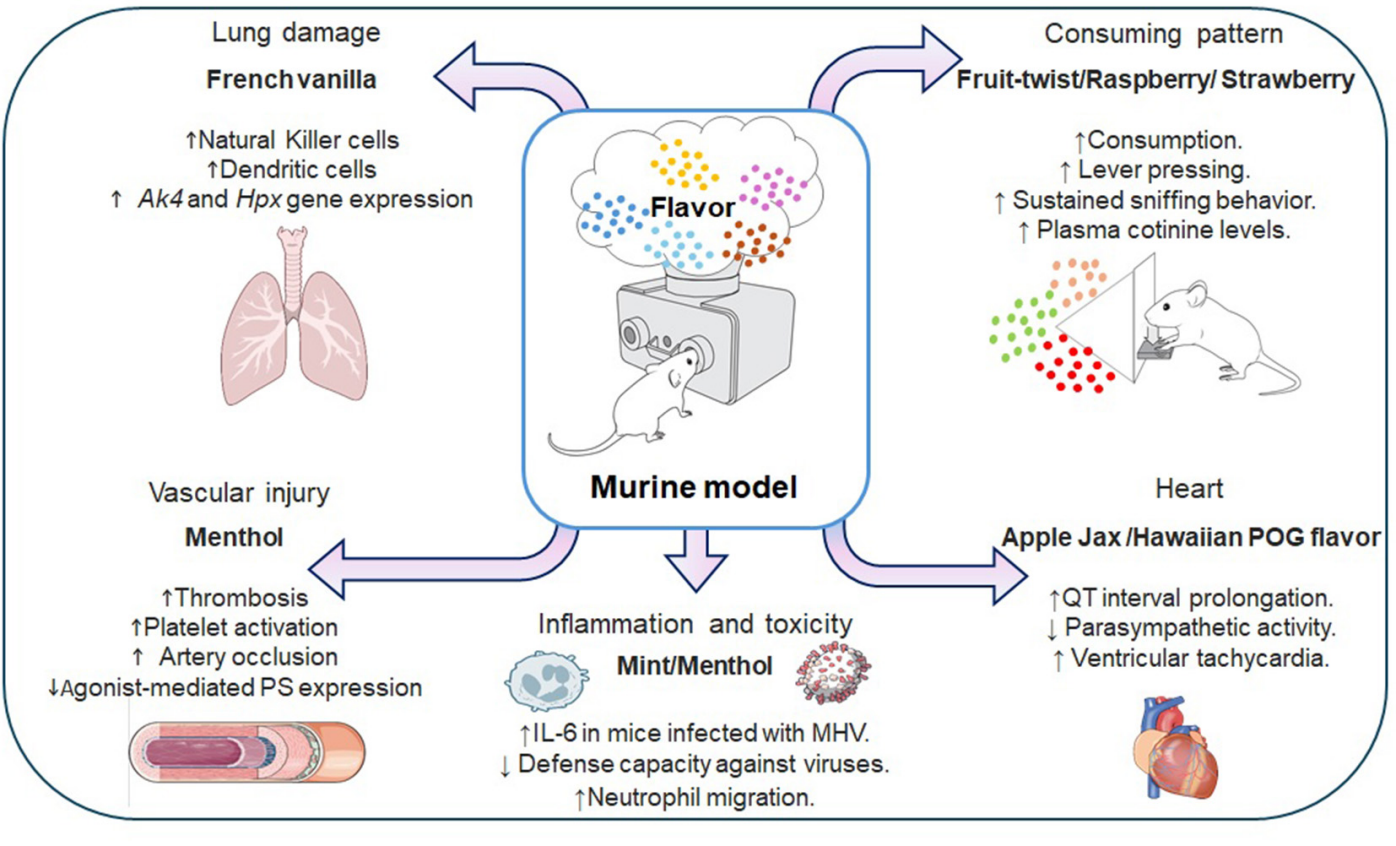
Organic and systemic effects of the use of flavored electronic cigarettes in mouse models. AK4, Adenylate kinase 4; HPX, Haemopexin; PS, Phosphatidylserine; IL-6, Interleukin 6; QT interval, Represents the total time it takes to depolarize and repolarize the cardiac ventricles; MHV, Murine hepatitis virus. Made with: https://scidraw.io/, https://bioart.niaid.nih.gov/, and https://bioicons.com/.

Animal models have identified the impact of flavored e-cigarette use on several pathways, including those associated with the immune system, oxidative stress, and DNA damage, among others. These findings reinforce what has been previously reported *in vitro* models, providing further evidence that flavored e-cigarettes, regardless of the presence or absence of nicotine, could cause harm to users' health, especially during chronic use (Figure 2).

### Reports on health risks associated with flavored e-cigarette users

Vaping-associated acute lung injury (EVALI)-like symptoms have been reported among e-cigarette users whose vehicles contain, among other substances, nicotine and flavorings. Among Caucasian e-cigarette users, 33% reported experiencing some EVALI-like symptoms, including cough, nausea, dry mouth, headache, or bad breath. Additionally, 87.3% reported using a flavored product, with the most common flavors being fruit (43.5%), mint/menthol (30.2%), candy/dessert (30.5%), and candy (24.8%) (34).

Although studies on e-cigarettes and their health effects remain limited, evidence suggests that using these products is not safe (Table 2). Chemical analysis of exhaled vape emissions has shown that flavors such as mint, watermelon, apple, vanilla, and mango generate high concentrations of VOCs, including acetaldehyde, acetone/propanal, acrolein, butane, and formaldehyde (35).

**Table 2:** Findings on flavored e-cigarette users.

Age (years old)	Female (%)	Vaping time (years)	Flavor	Device type	Findings	Reference
Greater than 21	NS	>1	Mint, watermelon, apple, vanilla, and mango	Closed and open	In participants using open devices, VOC concentrations were an order of magnitude higher than those using closed devices. Increasing the wattage applied to the vaping device's coils correlated with a higher total VOC production. Participants using open devices inhaled and absorbed higher doses of VOCs than those using closed devices.	Hopstock et al. (35)
45–80	55.3	≤ 1	Menthol	Closed	After adjusting for age, gender, race, pack-years of smoking, and the use of nicotine-containing vaping products, an association was identified between menthol use and reduced lung function. On average, menthol users had a 9.6% lower FEV1 than users of other flavored products.	Chandra et al. (36)
24.3	16.7	2.9 ± 0.4	Fruit, sweet, mint, menthol	Closed	E-cigarette users exhibited a 2.6-fold increase in DNA damage. Those who vaped sweet-flavored liquids displayed the highest DNA damage in oral cells compared to non-users, followed by those who used mint/menthol-flavored and fruit-flavored liquids.	Tommasi et al. (37)
50	100	0	Strawberry-vanilla	MEADS^*^	The flavored aerosol resulted in a significant increase in blood oxygen level-dependent signals in five brain regions: the right orbitofrontal and insular cortices, bilateral frontal operculum and frontal pole, left superior parietal lobule, precentral, postcentral, and supramarginal gyri, along with the left cerebellum and occipital fusiform gyrus.	Hobkirk et al. (38)

VOC, volatile organic compounds; FEV_1_, forced expiratory volume in the first second; MEADS, Magnetic Resonance Imaging Electronic Aerosol Delivery System.

Menthol is one of the most commonly consumed flavors; however, adding menthol to the base of freshly prepared e-liquid (propylene glycol and vegetable glycerin) improves particle counts across all size fractions tested, similar to the effect of adding vitamin E acetate. A retrospective analysis of menthol-flavored e-cigarette users from the COPDGene study showed a 9.6% reduction in forced expiratory volume in 1 s (FEV1) and a 0.06% reduction in FEV1/FVC, compared with users of non-menthol e-cigarettes. These findings suggest that this flavor not only increases exposure to harmful particles but also impairs lung function (36). Users of sweet-flavored e-liquids showed the highest levels of DNA damage in their oral cells, with a 1.26-fold increase compared to non-users (37).

At the neuronal level, a functional magnetic resonance imaging study analyzed the effects of flavored aerosols on brain activation in nine female daily smokers. Strawberry and vanilla flavored sprays reduced activation in reward regions compared to unflavored sprays. These flavors stimulated areas that mediate taste perception, such as the orbitofrontal cortex and the insula. Furthermore, greater functional connectivity was identified between subcortical and cortical regions, potentially reinforcing addictive circuits. Interestingly, brain activation with unflavored aerosols correlated with greater nicotine dependence and cravings, suggesting that sweet flavors may mask the immediate effects of nicotine while facilitating the development of addictive behaviors in the long term (38).

**Figure 3:**
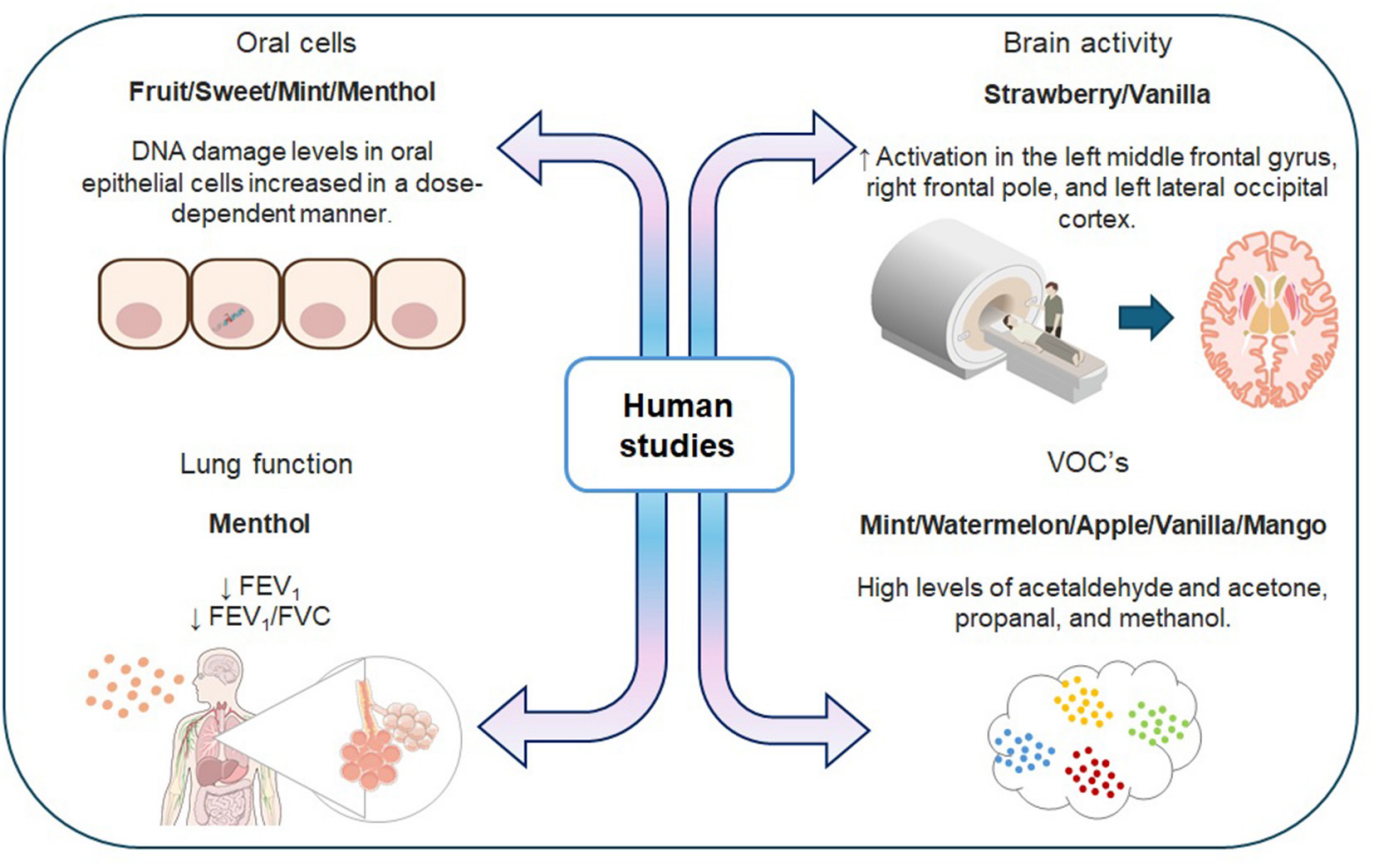
Biological impact of the use of flavored electronic cigarettes in humans. FEV1, forced expiratory volume in the first second; FVC, forced vital capacity; VOC's, volatile organic compounds. Made with https://scidraw.io/, https://bioart.niaid.nih.gov/, and https://bioicons.com/.

These findings indicate that electronic cigarettes are as harmful as combustible cigarettes, and their use is not recommended, especially among young people (Figure 3).

## Discussion

The use of e-cigarettes has emerged as a global public health issue due to the complications they cause in the respiratory, vascular, and other systems (39, 40). It is well known that nicotine is a substance involved in the addiction mechanism, resulting in changes in the brain. This systematic review presents, with the evidence collected to date, that the flavors used in electronic cigarettes are not inert and, after chronic use, could pose health risks to users. It has been observed that flavors influence acceptance and increase e-cigarette use (41–43). Pleasant flavors like menthol and fruity notes can make high nicotine levels more tolerable, decreasing the initial aversion to e-cigarette use (44). Flavor perception is a multisensory experience involving smell, taste, and touch. In vaping, this perception is primarily retronasal, as airflow from the back of the mouth and pharynx enhances the overall sensory response. This explains the preference for flavors among e-cigarette users (45).

The e-liquid market has grown increasingly complex, now offering nearly 250 flavors (46). This complexity is compounded by the increasing tendency among e-cigarette users to mix liquids. Among these, the most common flavors are fruit (23.9%), dessert (15.6%), candy (10.1%), menthol/mint (3.7%), coffee/tea (2.8%), and alcoholic beverages (1.8%) (47).

These devices pose a scientific challenge due to their continuous technological evolution. Each new generation of e-cigarettes introduces changes in aerosol delivery methods, thereby modifying their cellular and physiological effects. As a constantly evolving technological product, e-cigarettes pose new challenges for standardizing how we study them, since changes in their systems can lead to variations in the emission of harmful substances (43).

Fruity and menthol flavors in closed systems contain lead (Pb), tin (Sn), and antimony (Sb) particles. For Pb, the average lead emission is 377 μg/kg for fruit flavors and 150 μg/kg for menthol flavors (48). Open systems, due to the contact of the e-liquid with the metallic components of the device, particularly the heating resistors, can release heavy metals such as iron (Fe), arsenic (As), and nickel (Ni). For Ni in aerosol, it had an average presence of 1,181 μg/kg for fruit flavor (2).

Animal studies have shown that exposure to e-cigarette liquids and aerosols can cause significant neurobiological, respiratory, cardiovascular, and developmental harm. Reported effects include neuronal damage, embryonic malformations, delayed neuronal development, and disruption of the retinoic acid axis. Cardiovascular changes, including arrhythmia due to fluctuations in blood pressure and heart rate, have also been reported. However, methodological heterogeneity makes it difficult to establish results derived from standardized methods. Some studies used direct exposure of animal models to the flavored liquid, while others used exposure to aerosols, like what the user would do. In this regard, international guidelines, such as those proposed by the Center for Cooperation in Scientific Research on Heated Tobacco Products (CORESTA), can provide a valuable framework for future research standardization (49). However, the wide variety of nicotine delivery devices on the market, along with the flavors, increased the complexity of determining exposure to the flavors present in the studied animal model. In the case of flavored liquids, labeling problems were detected even in commercial products, prompting researchers to prepare their own mixtures and, in some instances, determine the actual concentrations of HPHCs.

The 75% of the studies included in this review were of high quality according to the Newcastle-Ottawa scale. There is limited evidence exploring the potential consequences for users of flavored e-cigarettes. One of the most notable limitations in human studies was the small sample sizes across studies and the exposure method. The study subjects were exposed to aerosols in a controlled manner, a situation that markedly differs from the everyday life of e-cigarette users. However, available evidence indicates that the use of flavors in e-cigarettes has significant adverse effects on human health, even in the absence of nicotine. Therefore, based on the evidence gathered, the damage resulting from the consumption of flavored electronic cigarettes can affect different organs such as the heart, lungs, and brain.

## Conclusions

Flavored e-cigarettes, after chronic use, could cause significant biological changes in users. These findings demonstrate that their use poses a potential health risk comparable to that of conventional tobacco cigarettes, which should be explored in more detail following previously standardized guidelines.

## Data Availability

The original contributions presented in the study are included in the article/Supplementary material, further inquiries can be directed to the corresponding author.
