# Supplementary material for: Findings on e-cigarette flavors and their implications for human health: a systematic review
Source: Front Public Health. 2026 Apr 30;14:1807715. doi: 10.3389/fpubh.2026.1807715 (PMC13171489; doi:10.3389/fpubh.2026.1807715)
Supplement: Supplementary file 1 [file Data_Sheet_1.docx]

Figure 1. PRISMA 2020 flow diagram for new systematic reviews, which included only database and register searches.

**Identification of studies through databases and registries**

Records removed prior to screening:

• Duplicate records (n = 189)

• Records flagged as unsuitable by automation tools (n = 0)

Conflict of interest: 19

Cellular study models: 21

In silico studies: 5

Cannabis cigarette, nicotine pouches and waterpipe trials: 4

Not meeting the review objective: 178

Records identified from:

PubMed, n = 225

Cochrane, n=205

**Identification**

Records screened

(n = 14)

Records excluded (n= 1)

The animal model was focused on nicotine effects, not flavours.

Reports not retrieved

(n = 0)

Reports sought for retrieval

(n = 0)

**Screening**

Reports assessed for eligibility

(n = 13)

Reports excluded: (0)

Studies included in review

(n =13)

Animal models: 9

Human studies: 4

**Included**

Modified from Page MJ et al. BMJ 2021;372:n71. doi: 10.1136/bmj.n71.

Table 1 supplementary

| SYRCLE risk of bias assessment of included studies | | | | | | | | | | |
| --- | --- | --- | --- | --- | --- | --- | --- | --- | --- | --- |
| Author (Reference) | Selection bias | | | Performance bias | | Detection bias | | Attrition bias | Reporting bias | Other |
|  | Sequence generation | Baseline characteristics | Allocation concealment | Random housing | Blinding | Random outcome assessment | Blinding | Incomplete outcome data | Selective outcome reporting | Other sources of bias |
| Wong (25) |  |  |  |  |  |  |  |  |  |  |
| Patten (27) |  |  |  |  |  |  |  |  |  |  |
| Ramirez (30) |  |  |  |  |  |  |  |  |  |  |
| Szafran (31) |  |  |  |  |  |  |  |  |  |  |
| Abouassali (32) |  |  |  |  |  |  |  |  |  |  |
| Marusich (26) |  |  |  |  |  |  |  |  |  |  |
| Alshareef (28) |  |  |  |  |  |  |  |  |  |  |
| Onyenwoke (29) |  |  |  |  |  |  |  |  |  |  |
| Dickinson (33) |  |  |  |  |  |  |  |  |  |  |
|  | |  |  |  |  |  |  |  |  |  |
| Low risk | |  |  |  |  |  |  |  |  |  |
| Unclear risk | |  |  |  |  |  |  |  |  |  |
| High risk | |  |  |  |  |  |  |  |  |  |

Table 2 supplementary

| The Newcastle–Ottawa Scale collection sheet | | | | | | | | | | |
| --- | --- | --- | --- | --- | --- | --- | --- | --- | --- | --- |
|  | Clearness of the Aim (0-2) | Sample Selection | | | | Comparability | | Outcome | | NOS Total Score (Quality Assessment) (0–16 |
| Author (Reference) |  | Representativeness (0-2) | Sample Size (0-2) | Non-Response Rate (0-2) | Exposure Assessment (0-2) | Control of Confounding Factors (0-1) | Comparability of Groups (0-1) | Assessment of the Outcome (0-2) | Statistics (0-2) |  |
| Hopstock (35) | 2 | 1 | 1 | 2 | 2 | 0 | 0 | 2 | 0 | 10 |
| Chandra (36) | 2 | 2 | 2 | 2 | 2 | 1 | 0 | 2 | 1 | 14 |
| Tommasi (37) | 2 | 2 | 2 | 2 | 2 | 1 | 1 | 2 | 2 | 16 |
| Hobkirk (38) | 2 | 1 | 2 | 2 | 2 | 1 | 0 | 2 | 2 | 14 |
